# Microbeam X-ray and Scanning Electron Microscopic Analyses on Sector-Banded Spherulites of Poly(p-dioxanone) Justified with Pixelated Iridescence

**DOI:** 10.3390/polym16192736

**Published:** 2024-09-27

**Authors:** Eamor M. Woo, Chia-Hui Lin, Selvaraj Nagarajan, Chean-Cheng Su

**Affiliations:** 1 Department of Chemical Engineering, National Cheng Kung University No. 1, University Road, Tainan 701, Taiwan; j6834006@gmail.com (C.-H.L.); nagarajan.tech@gmail.com (S.N.); 2 Department of Chemical and Materials Engineering, National University of Kaohsiung, No. 700, Kaohsiung University Rd., Nan-Tzu Dist., Kaohsiung 811, Taiwan; ccsu@nuk.edu.tw

**Keywords:** poly(p-dioxanone), poly(p-dioxanone), microbeam X-ray diffraction, periodic assembly mechanisms, iridescence

## Abstract

Poly(p-dioxanone) (PPDO) is crystallized with amorphous poly(p-vinyl phenol) (PVPh) and tannic acid (TA) as co-diluents to regulate and induce dendritic-ringed PPDO spherulites, with spoke- or sector-bands, aiming for convenience of analyses on interior lamellar assembly. Morphologies and interior lamellar arrangement leading to the peculiar rings on individual dendrites are evaluated by using polarized-light microscopy (PLM) and scanning electron microscope (SEM). Combinatory microbeam small-/wide-angle X-ray scattering (SAXS/WAXS) analyses further confirm the unique assembly patterns in periodic cycles. Alternate gratings are packed with periodic ridges composed of feather-like branches and the valley is featured with some embossed textures. The periodic gratings in the ringed spokes resemble those in nature’s structured coloration and are proven to display light-interference iridescence.

## 1. Introduction

Polymers are known to crystallize into higher-hierarchical morphologies of various crystal-aggregated patterns known as spherulites. By definition, a spherulite is a ball-shaped crystal entity; in reality, it may assume a variety of non-ball shapes that are governed by temperature, diluents/impurities, super-cooling, confinement in thin film or bulk, etc. Polymer chains are known to chain-fold and self-align in nanometer crystal units known as “lamellae”, whose orientation results in optical birefringence to produce a variety of colored/retarded patterns when viewed in polarized-light microscopy. These lamellae, numbered in thousands, then self-aggregate into micrometer spherulites that may assume a variety of shapes dictated by controlling thermodynamic and kinetic factors imposed during nucleation and growth. Common shapes are disc-like with ringless or ring-banded patterns (alternate retardation bands when viewed in PLM), tree-like dendrites [1,2,3], feather-like dendrites [4], hexagons [5,6,7,8,9], sea-weed-like [10,11], or sometimes multiple-facets co-sharing the same nucleus center (Janus-face, or sector-face) known as composite-sectored spherulites [12,13,14], etc. Crystalline morphology with periodic assembly has drawn the attention of researchers for a long time. Classical interpretations were mainly based on chain-folding-induced stresses being the origin for lamellar twisting [15,16,17,18,19,20,21,22,23,24]; yet, there are newer and more fitting models to account for such periodic crystal self-assembly [25,26,27]. As conventional studies mostly and mainly focused on typical circularly banded spherulites of polymers; this investigation initiated a new direction by examining periodic crystals that are uniquely different from the typical circular spherulites.

Poly(p-dioxanone) (PPDO) is chosen as this research work’s model target due to its distinguished biodegradability, biocompatibility, and versatile bio-medical material applications [28,29,30,31,32,33]. PPDO is a ductile poly(ether-ester) material widely used as bio-medical sutures, bone fixtures, surgery appliances, etc. [34,35,36,37]. Unlike biodegradable polyesters that are usually rather brittle, PPDO finds applications as surgery fixtures [36], tissue materials, bone repairs, etc. [37]. Our previous work has demonstrated that as PPDO is blended with H-bonding materials, such as poly (vinyl phenol) PVPh [1,38,39,40,41] or tannic acid (TA) [42,43,44,45], the PPDO assembled morphology can be tailored to a variety of patterns. Many researchers’ past investigations had merely focused on bio-degradability; alternatively, in terms of morphology, only on top-surface morphology analyses; the interior lamellae-assembly structures and crystallization growth mechanisms are yet still deficient. Geometric shapes of spherulites may influence macroscopic mechanical properties of polymeric materials, such as tensile strength and Young’s modulus, with crystallinity being one of the major determinants [46].

The main aim of this study was to analyze the top surface topography in correlating with the interior lamellar arrangement of the ring-banded spherulites of PPDO via 3D dissection of interior morphology. Novel approaches are coupled with powerful synchrotron–radiation X-ray analyses with microfocal techniques. PPDO is known to strongly interact with poly(p-vinyl phenol) (PVPh) [1,38,39,40,41] or tannic acid (TA) [42,43,44,45] via H-bonding, leading to miscible blends. Poly(vinyl alcohol) (PVA) is also known to interact with PPDO via H-bonding to induce peculiar morphology [47]. For the purpose of regulating the crystalline morphology of PPDO, two amorphous constituents, PVPh and TA, were simultaneously added as co-diluents, in various fractions, for modulation and regulating the crystalline morphology for convenience and accuracy of the interior architectures. Microbeam small-/wide-angle X-ray scattering (SAXS/WAXS) is used to verify the internal lamellae’s micro-structures and orientation. Tests of the photonic iridescence properties of PPDO were performed to support the grating-like periodic assembly.

## 2. Experimental

### 2.1. Materials and Preparation

**Scheme 1:**
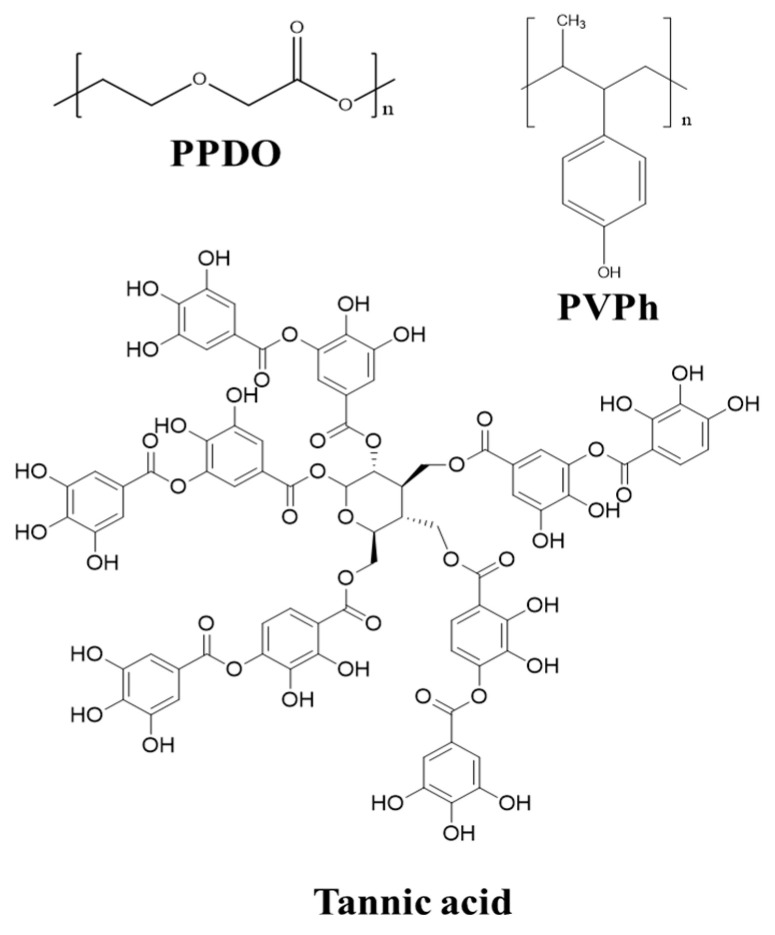
Chemical structures of PPDO, PVPh, and tannic acid.

PPDO was purchased from Sigma-Aldrich, Inc. (St. Louis, MO, USA), with viscosity = 1.5–2.2 dL/g as measured by dissolving in hexafluoroisopropanol in 0.1% (*w*/*v*), T_g_ = −9.5 °C and T_m_ = 104.8 °C. PVPh was purchased from Polysciences, Inc. (Warrington, PA, USA), with M_w_ = 22,000 g/mol, T_g_ = 150.5 °C was purchased from Polysciences, Inc. (Warrington, USA), with M_w_ = 1720 g/mol, T_g_ = 38 °C. PPDO, PVPh, and tannic acid (TA) were co-dissolved into a common solvent of p-dioxane and made into a 4 wt.% polymer solution. Chemical structures were given in Scheme 1. Dried film samples were heated to T_max_ for 1–2 min on a hot plate to erase the thermal history, then quickly removed from the hot plate to a temperature-preset microcopy hot stage at specific isothermal crystallization temperatures till maximum crystallinity.

### 2.2. Apparatus

Differential scanning calorimetry (DSC). A differential scanning calorimeter (Perkin-Elmer, Diamond DSC, Shelton, CT, USA) was used, which is equipped with a mechanical intra-cooler for quenching. For characterization of the glass transition temperature (T_g_), samples were first quickly heated from −50 °C to 200 °C, held for 5 min to eliminate any thermal history, and then scanned at a heating rate of 20 °C/min. A continuous nitrogen flow in the DSC sample cell was maintained.

*Polarized-light microscopy (PLM and LM).* PLM was equipped with an automatic exposure device (Nikon Optiphot-2, Tokyo, Japan), a heating stage (Linkam THMS-600, Salfords, UK), a temperature control device (Linkam T95, Salfords, UK), and a digital image capture control system (Nikon Digital sight DS-U1, Tokyo, Japan). The samples were observed with 4×, 10×, and 40× objective lenses with calibrated image scale to observe and capture images in 200–800× magnifications. When necessary, an oil-contact lens was used to achieve a maximum magnification up to 2000×, where the objective lens was designed to work with a drop of oil (i.e., instead of regular air contact), making contacts between the lens and specimens on glass slides increase the resolving power of a microscope.

*High-resolution field-emission scanning electron microscopy (HR-FESEM)*. Scanning electron microscopy (Hitachi SU8010, Tokyo, Japan) was used for characterizing the fractured and top surfaces of the samples. The crystallized samples were pre-cut on the back of the glass slide with a diamond knife and broken along the pre-cut line after dipping in a liquid nitrogen environment to prevent uneven cross-sections. The samples were coated with platinum using vacuum sputtering (10 mA, 300 s) prior to SEM observation.

*Microbeam small-angle X-ray scattering/wide-angle X-ray diffraction (abbr.: mSAXS/mWAXS).* Synchrotron-radiation microbeam small-angle X-ray scattering and microbeam wide-angle X-ray diffraction measurements were performed at the beamline TPS-25A of the National Synchrotron Radiation Research Center (NSRRC, Hsinchu, Taiwan). The X-ray photon energy is 15 keV, equipped with a detector with a distance of 5.09 m for SAXS and 0.136 m for WAXS. Other parameters had a wavelength of 0.8251 Å, a q-range between 0.00292 and 0.33545 Å^−1^ for SAXS, and a range between 0.0629 and 3.53 Å^−1^ for WAXS. The microbeam size was ca. 5 × 7 μm^2^. WAXS data were used to analyze the specific location/orientation of the crystal plane, while SAXS data were used to analyze the lamellae orientations corresponding to the ridge and valley zones.

## 3. Results and Discussion

### 3.1. PPDO/PVPh Ring-Banded Spherulites

**Figure 1:**
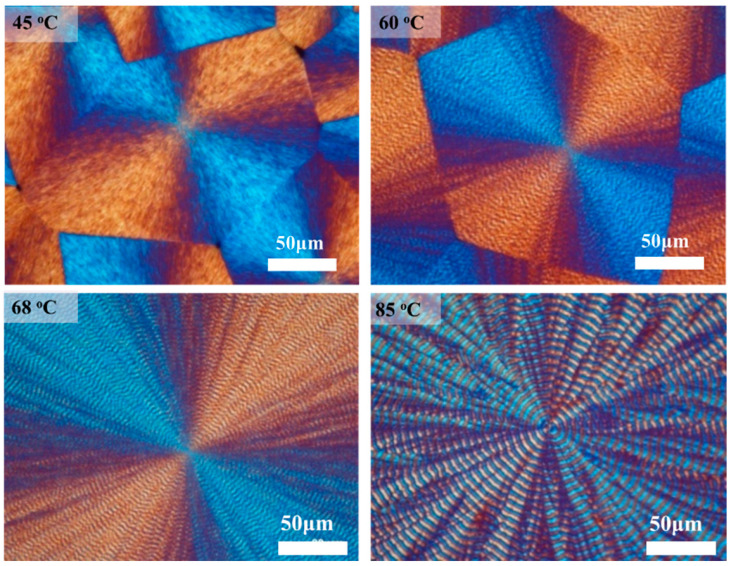
PLM graphs of spoke-banded PPDO crystals from the PPDO/PVPh (90/10) blend at increasing T_c_ = 45, 60, 68, and 85 °C, displaying a transition from being ringless and ring-banded to finally, being spoke-banded at increasing T_c_s.

Periodically banded spherulites in polymers are not uncommon; however, most of them are circularly smooth rings. Rings individually confined in discrete spokes are sometimes observed [32]. Figure 1 shows PLM graphs of PPDO crystals from a PPDO/PVPh (90/10) blend at increasing temperatures of T_c_ = 45, 60, 68, and 90 °C, displaying a transition from being ringless and ring-banded to finally being sector-dendrite-banded at high T_c_. For the PPDO crystals grown from the PPDO/PVPh (90/10) blend, they display a varying trend of transitioning from ringless, then smooth-circular rings, and finally to zig-zag spoked ring bands with respect to increasing T_c_. The ring bands at high T_c_ are no longer circularly smooth but spread in separate spokes, resembling cactus arms.

It is known that the assembly patterns in polymer spherulites can undergo a systematic transition with respect to variations in temperature. Typically, periodic bands are seen within a limited temperature window; outside the window, either ringless or other corrupted patterns are present. Figure 1 shows PLM graphs of PPDO spherulites displaying a transition from ringless at low T_c_’s, circularly ring-banded at intermediate T_c_’s, and finally to spoke-banded at high T_c_’s. The “spoke-banded” pattern (e.g., PPDO at T_c_ = 85 °C) is defined as bands assembled on respective spokes of lamellae that radiate outward from a common center. PPDO crystallizes from the PPDO/PVPh (90/10) blend at low T_c_ such as 45 and 60 °C and develops ringless or corrupted ring bands. Only at T_c_ = 68 °C does it display circularly ordered bands. Notably, even at T_c_ = 68 °C, the ring bands may appear to be mostly smooth circular; however, upon closer inspection, there are subtle spoke interfaces. At high T_c_ = 85–90 °C, the spoke-ring pattern becomes vividly apparent. For reference in brevity, SI Figure S1 shows PLM graphs of PPDO spherulites crystallized from the binary PPDO/PVPh (90/10) blend crystallized at the wide range of T_c_ = 40–90 °C. One can see that at higher T_c_’s, the patterns have a consistent trend by transferring from ringless to circular bands and finally to spoke bands.

**Figure 2:**
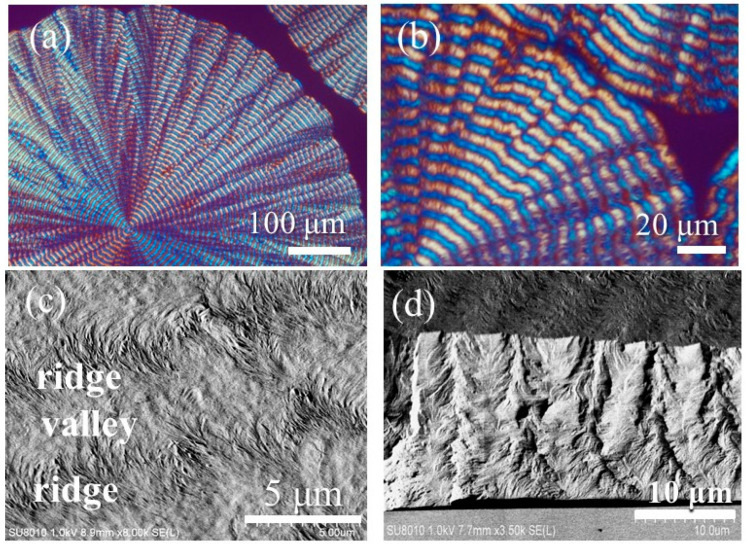
Spoke-bands in PPDO spherulites crystallized from PPDO/PVPh (90/10) at T_c_ = 88 °C: (**a**) PLM graph of a whole spherulite, (**b**) PLM zoom-in to individual spoke bands, (**c**) SEM zoom-in to nano-sized fibril dendrites on the top surface, and (**d**) interior dissection revealing periodic bands of the fractured specimen.

The interior dissection of individual spoke bands was performed using SEM characterization on fracture specimens. Figure 2a,b shows OM results where rings are present in individual spokes, named “spoke-bands”, in the PPDO spherulites crystallized from PPDO/PVPh (90/10) at T_c_ = 88 °C. Due to the spoked pattern, the rim of spherulites is not smooth-circular, but rather a flower’s multiple petals.

### 3.2. Spoke-Bands in PPDO Spherulites

**Figure 3:**
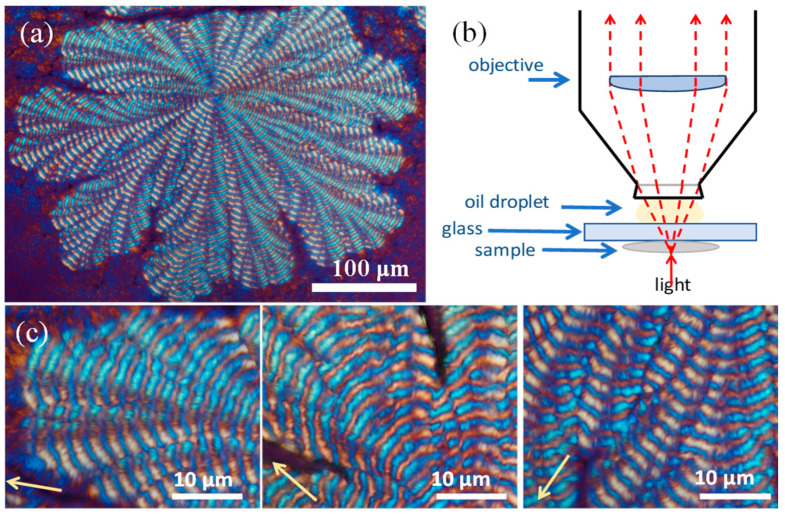
(**a**) PLM graph of the integral spherulite of the PPDO/PVPh/TA (80/10/10) blend crystallized at T_c_ = 78 °C, (**b**) PLM (oil immersion objective) operating principle, and (**c**) PLM graphs (oil immersion objective) of different locations in the PPDO/PVPh/TA (80/10/10) spherulite crystallized at T_c_ = 78 °C. [→ radial direction].

Oil-contact lenses allowed magnification up to 2000× in polarized-light microscopy. Figure 3a shows an entire spoked PPDO spherulite using a typical PLM. Figure 3b shows a scheme for setting up oil-contact in the microscope. By using an oil-contact lens, Figure 3c shows four images of four different zones of PPDO crystallized at T_c_ = 78 °C. This pattern is termed a “dendritic-ringed spherulite”, where individual ring bands are separately aligned in the spokes of the spherulite. This is dramatically different from the continuous ring bands on smoothly circular spherulites.

**Figure 4:**
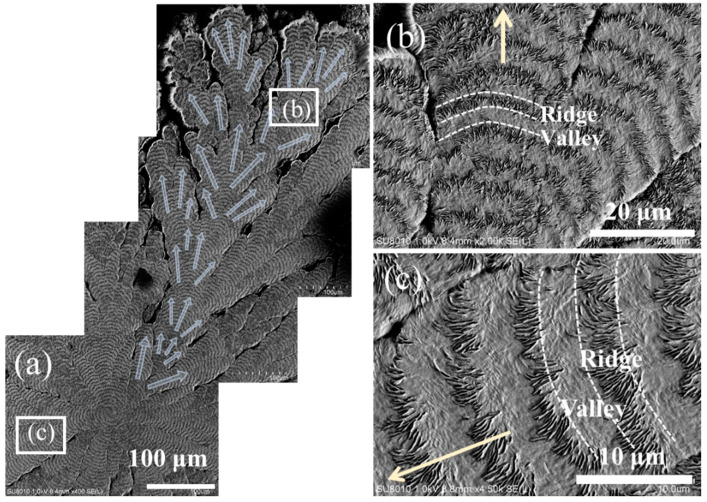
Sector-banded PPDO crystallized from a ternary PPDO/PVPh/TA (80/10/10) blend at T_c_ = 78 °C: (**a**,**b**) SEM micrograph of an arm on single spoke, (**c**) zoom-in white square-b to reveal the microscopic structure on the top surface. [arrows revealing the radial direction].

From the above OM graphs, it is obvious that periodic ring bands are present in each of the spokes of PPDO spoke-banded spherulites. SEM characterization was performed on a selected single spoke, whose morphological results are representative of those in all other spokes. Each spoke, on zoom-in, actually appears as a ginger root, showing several side arms at oblique angles to each other. A closer inspection of any of the arms would reveal that alternate valley ridge bands are present (Figure 4a,b). Figure 4c shows a stepwise zoom-in to greater magnifications. The ridge zone is populated with numerous fiber-like lamellae (feather-like fractal expansion) oriented in the radial direction, which are interfaced with a texture-less valley zone (Figure 4c). The circumferential-oriented valley zone appears to be packed with submerged fibrils oriented in the circumferential direction, appearing as an embossed pattern.

**Figure 5:**
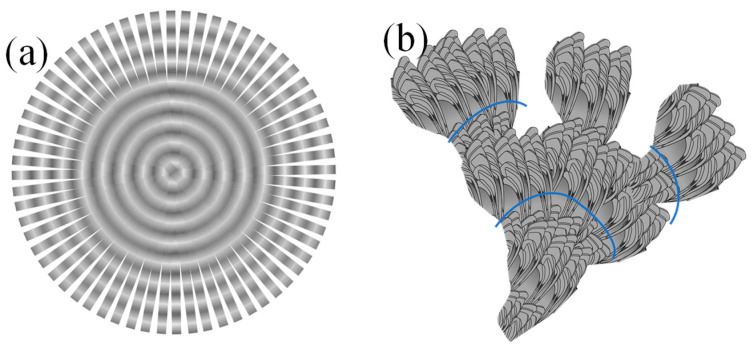
Schematics for growth of sector-banded spherulite: (**a**) regular smooth circular bands and (**b**) sector bands.

To emphasize the difference, Figure 5 shows the top surface of a common circular-banded vs. spoke-banded spherulite. Figure 5a is a typical circularly banded spherulite with ring bands in almost perfect concentric circles. By contrast, Figure 5b is a cartoon for the ginger- or cactus-like arms in a spoked band. Periodic bands on each of the cactus-like arms evolve as fractal branches. Figure 5b shows that the edge-on lamellae in the ridge zone are interfaced with the circumferential-oriented flat-on lamellae in the “valley” zone. The arms are at an oblique angle of ca. 30–35° from each other. Such split-arm growth with oblique branching angles is seen in most polycrystalline aggregates packed with branches. By contrast, the smooth circular-banded spherulites in Figure 5a can be viewed as straight spokes packed closely, with very few side arms. Other than this gross difference, the alternate bands are assembled in almost identical mechanisms, as exemplified in Figure 5b.

**Figure 6:**
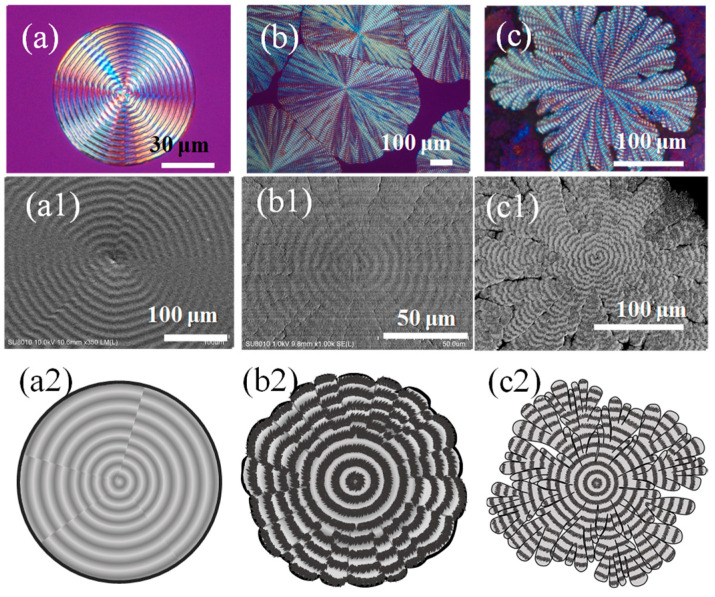
PLM, SEM images, and schematic diagrams showing a diluent-induced variation trend from smooth circular rings to spoke-segregated patterns, respectively, for (**a**,**a1**,**a2**) neat PPDO, (**b**,**b1**,**b2**) PPDO/PVPh (90/10), and (**c**,**c1**,**c2**) PPDO/PVPh/TA (80/10/10) spherulites crystallized at T_c_ = 78 °C.

Variation trends of PPDO morphology with composition changes are monitored to illustrate the effect of diluents (PVPh and TA). Figure 6 shows PLM, SEM images, and schematic diagrams showing a diluent-induced variation trend from smooth circular rings to spoke-segregated patterns, respectively. The temperature of isothermal crystallization was fixed at T_c_ = 78 °C, but the composition of PPDO/PVPh/TA was altered in three combinations. Figure 6a,a1,a2 pertains to PLM, SEM, and the scheme for the spherulitic pattern of neat PPDO; Figure 6b,b1,b2 pertains to that of PPDO/PVPh (90/10); and Figure 6c,c1,c2 pertains of PPDO/PVPh/TA (80/10/10), respectively. Apparently, neat PPDO at T_c_ = 78 °C maintains a typical circularly banded morphology. With the introduction of PVPh (10%) in the PPDOP/PVPh blend, the morphology displays a spoke-banded pattern. Finally, as PPDO is crystallized from the ternary PPDO/PVPh/TA (80/10/10) blend, the bands remain but the spoked morphology is altered into an irregular cactus-like pattern, where bands are present on each of the multiple arms. The periphery of the spherulite is highly zig-zag due to the extremely uneven growth of individual arms.

**Figure 7:**
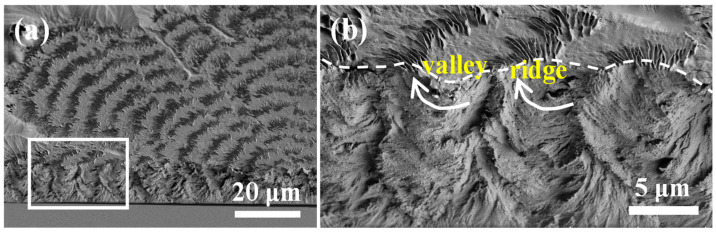
Spoke-bands in PPDO spherulite crystallized from the PPDO/PVPh/TA (80/10/10) blend at T_c_ = 78 °C: (**a**) SEM micrograph of the fractured interior vs. top surface in the radial direction and (**b**) zoom-in zone.

Interior dissection could shed light on the assembly mechanism. Delicate techniques of solvent-etching on specimens were adopted for exposing the interior assembly. The specimens, after fracturing, were etched using p-dioxane, prior to gold-sputtering and SEM characterization. The top surface and interior assembly could be correlated by positioning the specimens for SEM characterization by setting on sample stands at an oblique angle so that the electron beam could cover both fractured and top surfaces. Figure 7 shows SEM micrographs for one arm of the spoke bands in PPDO spherulite crystallized from the PPDO/PVPh/TA (80/10/10) blend at T_c_ = 78 °C. Figure 7a covers a wider zone of alternate bands in eight cycles at a lower magnification. Figure 7b is a zoom-in image at greater magnifications. Clearly, the interior lamellae are “U-shaped” if they are directly underneath the top-surface valley; conversely, they spring upward at the ridge bands and emerge as fibrils in the radial direction upon reaching the top surface to appear as the “ridge band”. Cycles self-repeat to pack in the same manner to appear as “bands” on the top surface; and due to the alternate orientation changes in lamellae in the interiors, PLM graphs display standard tint-color rings.

**Figure 8:**
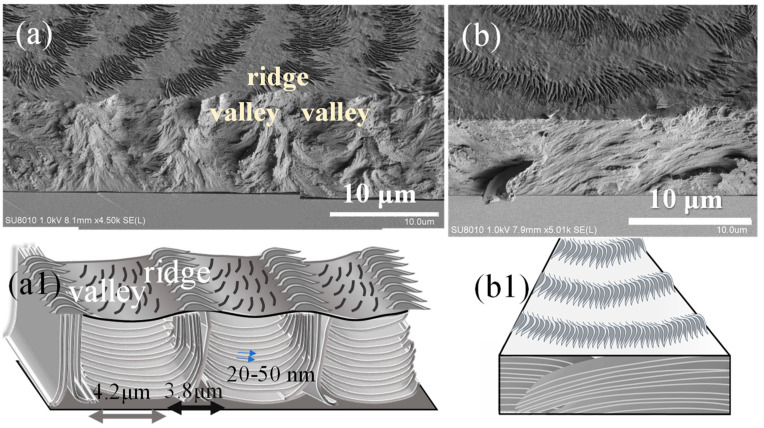
SEM micrographs of the fractured surface of spoke-banded PPDO spherulite crystallized from a PPDO/PVPh/TA (80/10/10) blend at T_c_ = 78 °C: (**a**) fracture along the radial direction, (**b**) fracture along the tangential direction, and (**a1**,**b1**) schemes depicting the top-vs.-interior fractured interiors corresponding to the lateral and front views, respectively. [→ radial direction].

Fractures of specimens were controlled to expose the interiors of individual spokes along the tangential and radial directions. SEM results are shown in Figure 8a (fracture along the radial direction) and Figure 8b (along the tangential direction). When cut in the radial direction, the interfaces between the successive bands become apparent, where fibril lamellae are clearly exposed (Figure 8a). When cut in the tangential direction, the top-vs.-interior correlation reveals that fibril lamellae of an oblique angle are clearly exposed to be located just underneath the top-surface valley zone (Figure 8b). To depict the respective morphological patterns, two schemes (Figure 8a1,b1) are used for the lateral view and front view, respectively, of the interior grating assembly. Obviously, the growth is self-repetitive in cycles with periodic interfaces and not a continuous helix radiating out from a common nucleus. Discontinuity does exist between the successive bands distinctly in cyclic repetition until reaching the periphery of the spherulites. Figure 8a1 shows three self-repetitive cycles of growth along the radial direction, where the bands discretely self-assemble in each of the radial-oriented spokes (or “cactus-like” arms). Notably, each of the three cycles is almost identical, and the sum of widths of the valley and ridge (4.2 and 3.8 μm) constitutes an inter-band spacing = 8 μm. The assembly in individual spokes is not unlike those seen in common circularly smooth banded spherulites that are widely reported in the literature.

### 3.3. Synchrotron Microbeam X-ray Analysis on Spoke-Segregated Ring Bands

**Figure 9:**
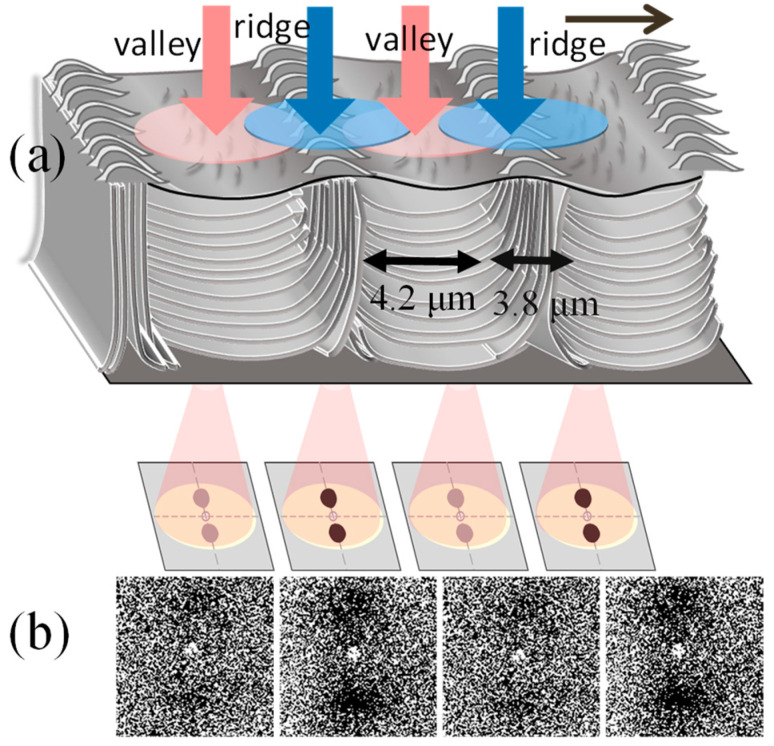
(**a**) Scheme for 3D interior assembly, and (**b**) microbeam 2D-SAXS patterns with respect to lamellar orientation changes in spoke-banded PPDO crystallized from the PPDO/PVPh/TA (80/10/10) blend at T_c_ = 78 °C [microbeam size ≈ 6 μm, wavelength = 0.8251 Å] [→ radial direction].

In addition to the 3D microscopic dissection evidence for revealing the assemblies in micro- to nano-scales, X-ray analysis could yield powerful support to the interior crystal assembly. To achieve this, microfocal X-ray beams from a synchrotron radiation facility were necessary, where the size of the beams could be adjusted to suit the narrow inter-band spacing to pick up the crystal orientation changes in micrometer ranges. Microbeam small-angle X-ray scattering (mSAXS) was performed on properly prepared specimens (cast on transparent PI films). The microbeam size is ~6 μm, which is sufficient for analyzing the inter-band crystal changes. Results are depicted in Figure 9a,b, where the scheme of Figure 9a illustrates when the microfocal beamline penetrates the ridge (top and interior). For better visualization, Figure 9a shows the 3D interior assembly of periodic architecture for the top surface and interior. In the scheme, the intended X-ray microbeam is positioned on specific spots that are cyclically on top of the valley and ridge zones as viewed on topology. Figure 9b shows the microbeam 2D-SAXS patterns with respect to changes in lamellar orientation in the PPDO/PVPh/TA (80/10/10) blend crystallized at T_c_ = 78 °C. To better illustrate the not-so-strong signals (due to thin film), a simplified scheme is drawn on top of the real SAXS signals. Apparently, as the microbeam is directed to be on top of lamellae that are parallel to the X-ray beam, there is virtually no signal (on the valley zone). Conversely, as the microbeam is step-moved to the valley zone where the interior lamellae are mostly perpendicular to the microbeam, there is a distinct dot-like twin signal on the meridian line. The microbeam was moved as shown in Figure 9a to complete two cycles of ridge-valley, and the SAXS signals repeat in two identical patterns, thus proving that the interior lamellae are indeed assembled in the same cyclic manner as that illustrated in the cartoon.

The above SAXS results can be further expounded via internal crystal lattices in the lamellae as the lamellae undergo periodic orientation changes. From the microbeam SAXS results, it is evident that the variations in the periodic arrangement or orientation of the lamellae plates can be emulated. In addition to microscopy dissection into interiors, the X-ray analysis confirms the discontinuous grid-like internal crystal plate arrangement. Consequently, for achieving a precise morphological comparison with the SEM morphology, the use of a delicately set up SAXS microfocal beam is necessary.

**Figure 10:**
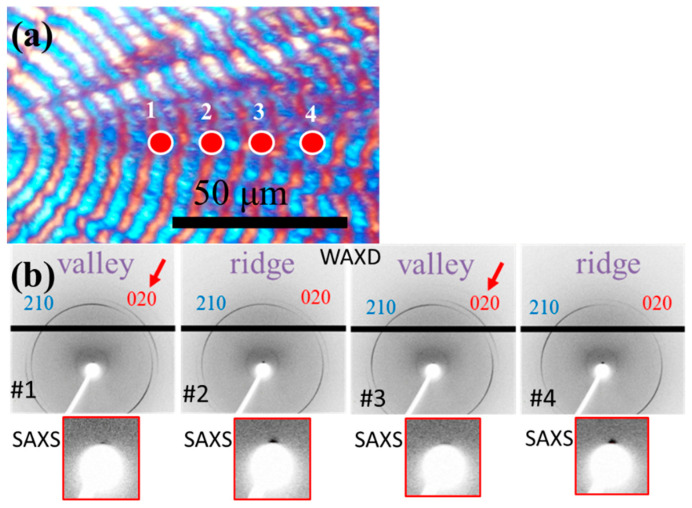
Microbeam 2D WAXS and SAXS patterns: (**a**) spots marked on the PLM graph of PPDO crystallized from PPDO/PVPh (90/10) at T_c_ = 78 °C corresponding to (**b**) microbeam WAXS and SAXS 2D patterns with step-moving from spot #1 to #4 [microbeam size ≈ 6 μm].

By using the advanced technique of mWAXS for further analyses, step-wise changes in the sequential orientation of the crystal lattices in lamellae might shed some light. The focus was placed on two representative crystal planes in the lattice, (210) and (020), as these two signals were the most pronounced. Notably, in a blend of PPDO with amorphous PVPh and/or TA, the lamellae might have been loosened but the crystal lattices of PPDO remained unchanged, which is of an orthorhombic crystal system. Figure 10a shows the PLM graph of PPDO spherulite on which the microbeam X-ray was step-moved. Spots-1, 3 are in the valley region; by contrast, Spots-2,4 are in the ridge region. Figure 10b shows four mWAXS 2D patterns corresponding to these four spots, respectively. One easily discerns that (210) signals are visible in the ridge and valley regions; the (020) signal is stronger in the valley region but is much weaker at the ridge. Notably, the microbeam technique with a properly adjusted beam-stopper position allowed us to obtain WAXS and SAXS signals simultaneously. This was performed by slightly adjusting the position of the beam-stopper relative to the microbeam on-site of the sample stand. Indeed, SAXS signals in addition to WAXS ones were recorded at the same scans; however, it is notable that due to the beam-stopper positioning, the SAXS signals could be recorded only in relation to the top one-half, with the bottom-half signal being mirror-symmetric. The simultaneous microbeam X-ray diffraction (SAXS and WAXS) confirms and supports the grating assembly of interiors vs. the top-surface valley/ridge alteration.

### 3.4. Pixelated Iridescence by Light Interference with Periodic Micro- or Nano-Structures

White optical light can interfere with orderly gratings tuned with suitable microstructures for diffraction. For structures with interference with white light, it is likely that they may induce attractive and intensive spectral coloration in the PPDO-RBS. Nature is known to evolve many biological species with diversified periodic nano- or micro-structures, blessed with capacities to interfere with light to produce iridescence coloration to perform intended biological functions. The wings of an insect, namely of the morpho-didius iridescent butterfly, are known to gleam a striking blue color [48,49,50,51]. Photonic characteristics for coloration from the cuticle scales of beetles were studied in detail by Bartl et al. [52]. Cellulose in crystalline structures can be governed by biological designs to serve structural as well as non-structural cosmetic coloration purposes. Pollia Condensata fruit’s skin [53] displays unusual spiral-stacked cellulose crystals of multi-layered assembly that lead to reinforced interference with light to display brilliant blue coloration. In synthetic chemistry, cellulose nanocrystals (CNC) can be tailored by acid hydrolysis to exhibit a peculiar resemblance to the natural chiral nematic properties [54,55,56]. Some birds display colors in feathers due to a combination of both biological pigments and structural coloration. Peacock feathers [57] have pigments in barbules jointly with periodic micro-structures in barbs for producing colorful iridescence. Inorganic minerals can also be made into photonic crystals either by biological genetic designs or by self-assembly such as opals (SiO_2_ crystalline micro-particles in assembled orders). Nacre’s layered nanostructure (CaCO_3_ crystals) is well known to generate pleasant shiny colors [58,59]. Synthetic block-copolymers may form nano-phase separation at the nanometer level to larger domains at ca. 150 to 290 nm, which are reported to produce wrinkled textures with a capacity of light interference into colors [60,61,62,63].

**Figure 11:**
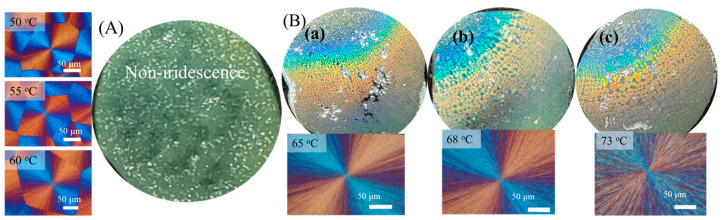
Non-iridescence vs. iridescence correlating with ring patterns and large size of PPOD spherulites crystallized from the PPDO/PVPh (90/10) blend: (**A**) non-iridescence at T_c_= 50 °C–60 °C (ringless) and (**B**) iridescence at T_c_ = (**a**) 65, (**b**) 68, and (**c**) 73 °C (all periodically banded).

Iridescence in nature may occur as a result of white light (sunlight) interacting with orderly assemblies that have aligned gaps in periodic bands with lamellar microstructures acting as gratings. For polymer crystals, such as what was reported in this work, assembled in orderly bands and iridescence due to light interference with orderly arrays can be expected and used as proof for lamellar assembly with periodic orders. Figure 11 shows (A) non-iridescence vs. (B-a,b,c) iridescence correlating with the ring patterns and size of PPOD spherulites crystallized from the PPDO/PVPh (90/10) blend. Obviously, when crystallized at 50 °C–60 °C, the PPDO spherulites are either ringless or display corrupted bands with no orderly gratings, and thus, they are non-interfering with light. When crystallized at T_c_ equal to or greater than 65 °C, the PPDO spherulites exhibit orderliness in ring bands and capacity for light interference (Figure 11B).

**Figure 12:**
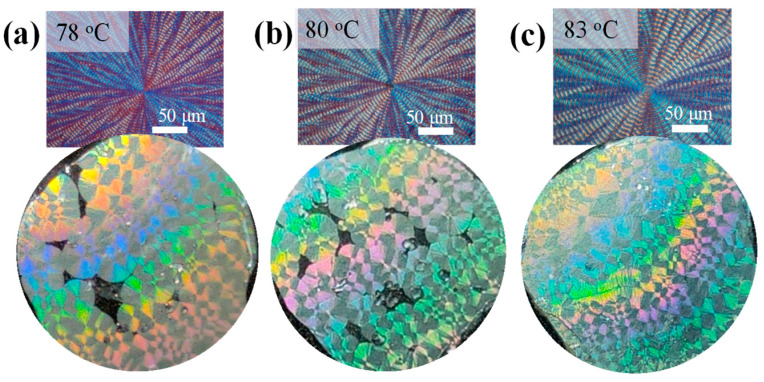
Pixelated iridescence coloration correlating with ring regularity and large-size PPDO spherulites crystallized from the PPDO/PVPh (90/10) blend at T_c_ = (**a**) 78, (**b**) 80, and (**c**) 83 °C (spoke-bands).

Figure 12 shows images of pixelated iridescence correlating with the ring regularity of the large-size PPDO bands crystallized from PPDO/PVPh (90/10) blend at T_c_ = (a) 78, (b) 80, and (c) 83 °C (spoke-segregated bands), respectively, where the crystallized spherulite sizes tend to be much larger. Iridescence has also been discovered in circularly smooth-banded assemblies of poly(p-dioxanone) (PPDO) or poly(butylene adipate) (PBA)-banded spherulites [64,65]. The strongly interacting PVPh diluent causes PPDO to display spoke-segregated bands, instead of common circularly smooth bands; nevertheless, orderliness is maintained in each of the individual spokes that are assembled with nano- to micro-structured arrays of parallel-grating lamellae capable of interfering with optical light. The orderly assembled PPDO spoked bands could also display similar traits of iridescence as those of typical circular banded PPDO or PB [64,65]. Brief and representative results are demonstrated for pixelated iridescence in PPDO/PVPh (90/10) spoke-banded crystals, where the large sizes are also responsible for the pixelated pattern of the spoke-banded PHBV spherulites crystallized at T_c_ = 78–83 °C.

**Figure 13:**
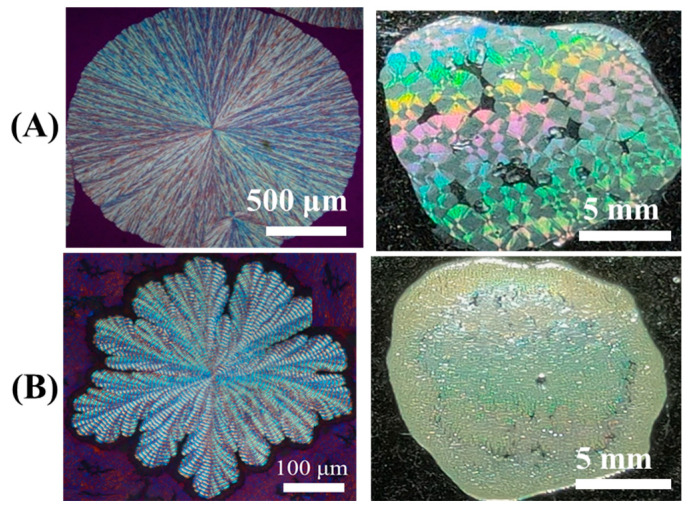
PLM for ring patterns (at T_c_ = 80 °C) and photonic iridescence images of (Row-**A**) PPDO/PVPh (90/10) blend in comparison with (Row-**B**) PPDO/PVPh/TA (80/10/10) blend.

The inclusion of additional diluents may further improve or disintegrate the ring regularity, which in turn impacts the light interference capacity. For proof, tannic acid (TA) was added into the PPDO/PVPh blend to form a ternary blend of PPDO/PVPh/TA = 80/10/10 (wt. ratio). PPDO spherulites were then crystallized from the ternary PPDO/PVPh/TA (80/10/10) blend at T_c_ = 80 °C. Figure 13 shows POM images for ring patterns (at T_c_ = 80 °C) of spoke-banded PPDO [from PPDO/PVPh (90/10) blend] vs. sector-banded PPDO spherulites [from PPDO/PVPh/TA (80/10/10) blend]. Both patterns are ring-banded; yet, spoke-banded PPDO maintains the regularity of grating structures but the sector-banded PPDO does not. As a result, photonic iridescence is seen in the spoke-banded PPDO spherulites (Row-A), but the sector-banded PPDO spherulites are non-iridescent (Row-B). The addition of TA resulted in a significant reduction in spherulitic sizes and disrupted the assembly orderliness of banding arrays.

## 4. Conclusions

By introducing amorphous and H-bonding PVPh and TA into PPDO, the crystalline PPDO morphology is induced to pack into loosened aggregates, with spoke-ring patterns. The interior-dissection results of the fractured morphology reveal orderly grating-assembled lamellae in both the interior and top surfaces. The interior lamellae of the spoke-banded PPDO are stacked more loosely than those of neat PPDO (with no diluents). By increasing the content of PVPh and TA, the morphology of the PPDO/PVPh/TA blend undergoes a systematic change from a smooth-circular ring-banded pattern to a highly spoked- or dendritic-ringed pattern. The results of the top surface show that the spherulite was composed of feather-like lamellae in the ridge zone and flat-embossed textures in the valley zone.

The correlation of assemblies of a top surface versus interior lamellar structure is established. From the results of the fractured surface to expose the interiors, the periodic assemblies are composed of two perpendicularly oriented lamellae, appearing as alternate ridge and valley, with edge-on lamellae in the ridge and flat-on ones in the valley, leading to an internal grating architecture. Fractured surfaces in the radial and circumferential directions were observed by SEM. The interior structure of spherulites shows that the flat-on lamellae stacked horizontally at the valley and grew upward from the substrate at the ridge. Synchrotron-radiation microbeam SAXS and WAXS analyze and verify the interior lamellae orientation observed by SEM. From the microbeam SAXS 2D patterns, the results demonstrate strong signals in the ridge corresponding to edge-on lamellae and weak signals in the valley corresponding to flat-on lamellae. The iridescence of periodically banded PPDO crystals has also been investigated, which is related to the internal grating architecture. Therefore, the 3D growth mechanism of ring-banded assembly and the lamellar arrangements of ring-banded PPDO custom-made by crystallizing from a ternary blend system could be revealed. Due to the diluent-tailored large-size crystals with parallel lamellae in orderly gratings, iridescence coloration is distinct and becomes pixelated patterns.

## Data Availability

The original contributions presented in the study are included in the article/Supplementary Material, further inquiries can be directed to the corresponding author.

## Supplementary Materials

The following supporting information can be downloaded at https://www.mdpi.com/article/10.3390/polym16192736/s1: Figure S1: PLM micrographs of PPDO crystallized from PPDO/PVPh (90/10) blend at T_c_ = 40–90 °C, at 5 °C intervals.
