# Supplementary material for: Microbeam X-ray and Scanning Electron Microscopic Analyses on Sector-Banded Spherulites of Poly(p-dioxanone) Justified with Pixelated Iridescence
Source: Polymers (Basel). 2024 Sep 27;16(19):2736. doi: 10.3390/polym16192736 (PMC11478648; doi:10.3390/polym16192736)
Supplement: Supplementary file 1 [file polymers-16-02736-s001.zip › polymers-3202730-supplementary.pdf]

Microbeam X-Ray and Scanning Electron Microscopic Analyses on Sector-Banded Spherulites of Poly(p-dioxanone) Justified with Pixelated Iridescence

Eamor M. Woo[1]*, Chia-Hui Lin[1], Selvaraj Nagarajan[1], and Chean-Cheng Su[2]

[1]Department of Chemical Engineering, National Cheng Kung University No. 1, University Road, Tainan, 701-01, Taiwan.

[2]Department of Chemical and Materials Engineering, National University of Kaohsiung, No. 700, Kaohsiung University Rd., Nan-Tzu Dist., Kaohsiung, 811, Taiwan. E-mail: ccsu@nuk.edu.tw

* Correspondence: emwoo@mail.ncku.edu.tw; Tel.: +886 6 275-7575 x 62670.

Supporting Info.

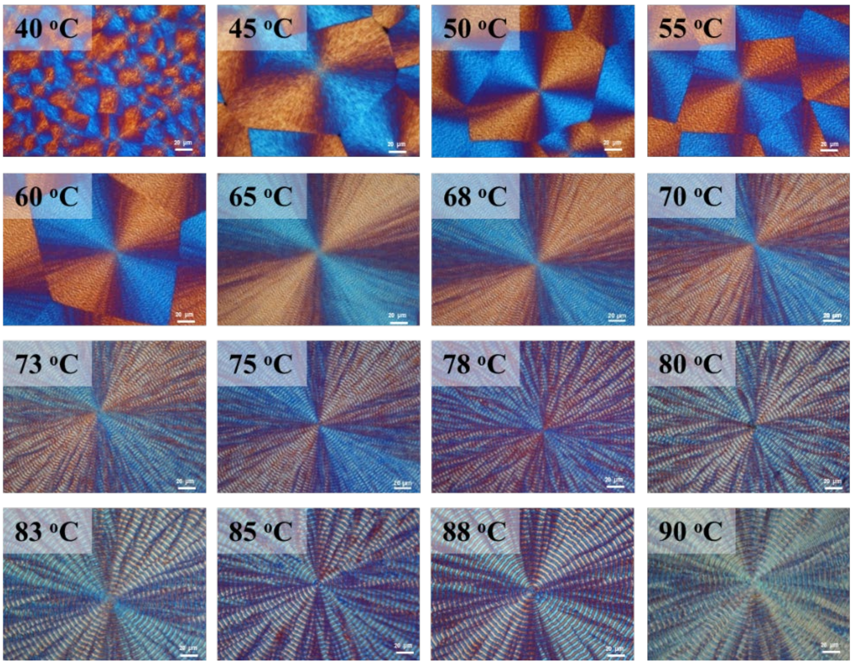

Figure S1. PLM micrographs of PPDO crystallized from PPDO/PVPh (90/10) blend at T_c = 40-90 °C, at 5 °C intervals.
